# Dual-Functional Solar-to-Steam Generation and SERS Detection Substrate Based on Plasmonic Nanostructure

**DOI:** 10.3390/nano13061003

**Published:** 2023-03-10

**Authors:** Ba Thong Trinh, Hanjun Cho, Deunchan Lee, Oleksii Omelianovych, Taehun Kim, Sy Khiem Nguyen, Ho-Suk Choi, Hongki Kim, Ilsun Yoon

**Affiliations:** 1 Department of Chemistry, Chungnam National University, Daejeon 34134, Republic of Korea; 2 Department of Chemical Engineering and Applied Chemistry, Chungnam National University, Daejeon 34134, Republic of Korea; 3 Department of Chemistry, Kongju National University, Gongju 32588, Republic of Korea

**Keywords:** dual function, gold nanoparticles, cellulose filter paper, solar-to-steam generation, surface-enhanced Raman scattering, water purification, pollution detection

## Abstract

Solar-to-steam (STS) generation based on plasmonic materials has attracted significant attention as a green method for producing fresh water. Herein, a simple in situ method is introduced to fabricate Au nanoparticles (AuNPs) on cellulose filter papers as dual-functional substrates for STS generation and surface-enhanced Raman spectroscopy (SERS) sensing. The substrates exhibit 90% of broadband solar absorption between 350 and 1800 nm and achieve an evaporation rate of 0.96 kg·m^−2^·h^−1^ under 1-sun illumination, room temperature of 20 °C, and relative humidity of 40%. The STS generation of the substrate is stable during 30 h continuous operation. Enriched SERS hotspots between AuNPs endow the substrates with the ability to detect chemical contamination in water with ppb limits of detection for rhodamine 6G dye and melamine. To demonstrate dual-functional properties, the contaminated water was analyzed with SERS and purified by STS. The purified water was then analyzed with SERS to confirm its purity. The developed substrate can be an improved and suitable candidate for fresh water production and qualification.

## 1. Introduction

Clean water is one of the most important components of life worldwide [1]. However, with the rapid growth of the human population and industrialization, water pollution is becoming more severe, and several organic compounds with high toxicity in water bodies, even at trace levels, can be harmful to human life [2,3]. Recently, direct solar-to-steam (STS) generation based on interfacial evaporation systems has been extensively investigated as an environmentally friendly solution for water purification [4,5,6,7,8,9,10]. Photons are first harvested by light absorbers and converted to thermal energy for water evaporation; then, steam generation occurs through surface evaporation at a temperature that is much lower than the boiling temperature of water (100 °C at 1 atmosphere pressure). [11,12]. Several types of materials are used to fabricate highly efficient light absorbers, such as plasmonic materials [13,14,15,16], carbon-based materials [17,18,19], polymers [20,21], and semiconductors [22,23]. Among them, plasmonic nanoparticles have the unique capability of controllable broadband light absorption and excellent heat conversion efficiency based on localized surface plasmon resonance [16]. Several plasmonic nanoparticles, including gold [14], silver [24], aluminum [25], copper [26], and nickel [27], which have suitable dielectric constants and strong surface plasmon resonances, have been utilized in interfacial water solar purification. Plasmonic gold nanoparticles are the most popular candidates owing to their excellent light-to-heat conversion efficiency [16,28]. Plasmonic materials have been utilized for surface-enhancement Raman spectroscopy (SERS) for applications in chemical detection, with significant improvements in the electromagnetic field when interacting with light [29,30,31,32]. The improvement in the electromagnetic field is attributed primarily to the nanogaps in the nanoparticle–hotspot region, where the electric field increases significantly when excited by a suitable wavelength [33,34]. Several studies have reported promising results for the SERS detection of organic pollutants based on Au nanostructures, such as dyes [10,35], pesticides [36], and food spoilage [37]. There have been many studies that have introduced plasmonic nanoparticles for STS generation [38,39,40] and water pollution SERS detection [41,42]. The combination of these two different functions using one material can significantly simplify the production and qualification of fresh water, which may be limited in outdoor places. Even though both STS generation and SERS detection using plasmonic nanoparticles are based on the interaction between the light and plasmonic nanoparticles, there are still differences in the required optical properties and structure designs. The absorbers for STS generation require broadband, high solar absorption, and a porous structure to facilitate mass-transports of liquid and vaporized water [4]. The SERS substrates usually require strong extinctions at the excitation laser wavelength and the Raman shift wavelength, and the flat substrates such as Si wafer or metal oxide film may be preferred for better SERS performance [43]. To bring these two functions into one structure, the most ideal design combines the plasmonic nanoparticles with a porous and hydrophilic material, as shown in some prior studies. Zhu et al. demonstrated an Ag-based asymmetric plasmonic structure for dual functions of solar water purification and pollutant detection [44]; reportedly, it exhibited an energy transfer efficiency of 80% in STS generation and ultra-sensitive chemical detection up to 100 pM. Li et al. used the porous fluorescent aerogel to adsorb exhausted Cr (VI) to apply in water purification and STS generation, with an evaporation rate of 1.31 kg·m^−2^·h^−1^ [45]. A self-assembling fluorescent hydrogel substrate was also utilized as a selective and sensitive sensor to detect Hg (II), providing a high evaporation rate of 1.30 kg·m^−2^·h^−1^, as shown a study by Li et al. [46].

In this study, Au nanoparticles (AuNPs) were reduced on cellulose substrate (AuNP/cellulose substrate) by a thermal process to fabricate a dual-functional solar water purification and SERS substrate. Cellulose filter paper was selected as the backbone of the substrate because of its hydrophilicity, rapid water transfer, flexibility, environmentally friendly disposition, and cost-effectiveness [47,48,49]. The AuNP/cellulose substrate was characterized to determine its morphologies and optical properties; subsequently, it was applied to a STS generation system under 1-sun illumination and typical environmental conditions. Next, the sensing properties of the substrate were investigated using water polluted with a common dye, rhodamine 6G (R6G), and melamine contaminant. Rhodamine 6G (R6G) is used as a colorant in the manufacturing of textiles and foodstuffs, and water or food contaminated with R6G is highly carcinogenic [50,51]. Melamine is a chemical compound with numerous industrial uses, including the production of plastics, dishware, kitchenware, and commercial filters, which can easily pollute drinking water and food [52]. Finally, the dual-functional substrate was employed for on-site water purification and pollutant detection experiments. 

## 2. Materials and Methods

### 2.1. Materials

Gold (III) chloride trihydrate (HAuCl_4_·3H_2_O, ≥99.9% trace metal basis), R6G (C_28_H_31_N_2_O_3_Cl, dye content 99%), melamine (C_3_H_6_N_6_, 99%), and sodium bicarbonate (NaHCO3, >99.5%) were purchased from Sigma-Aldrich (St. Louis, MO, USA). Nitric acid (HNO_3_, 70%) was purchased from Daejung Chemicals & Metals Co., Ltd. (Siheung-si, Republic of Korea). Commercial deionized water (18.25 MΩ, 25 °C) was purchased from Joylife Co. (Gimhae, Republic of Korea) All the chemicals were used without any further purification. Cellulose filter paper (Whatman #0905) was purchased from Cytival (Marlborough, MA, USA).

### 2.2. Synthesis of AuNP/Cellulose Substrate

First, the cellulose filter papers were cut into squares of 1.5 × 1.5 cm^2^ and cleaned with diluted HNO_3_ (10 *v*/*v*%) and NaHCO_3_ (10 wt%) for removing contaminates. After that, all the filter paper pieces were rinsed several times in deionized water to obtain the neutral pH condition and dried in the oven, then stored until use. Next, HAuCl_4_.3H_2_O (100 mM) dissolved in ethanol was employed as the Au precursor. Subsequently, different volumes of the precursor solution (30 µL, 40 µL, 50 µL, and 60 µL) were introduced to the pre-cleaned filter papers by drop-casting and dried at room temperature for 1 h. Subsequently, the samples were heated in an oven at 100 °C for 2 h. Next, the samples were rinsed, sonicated in DI water to remove all residual ions, dried in an oven, and stored until the experiments were performed.

### 2.3. Material Characterization

The surface morphologies of the samples were investigated using scanning electron microscopy (SEM, Tescan Clara, Czech Republic) with energy-dispersive X-ray spectroscopy (EDS) analysis. The substrates were sputtered with Pt for 60 s before SEM analysis via Ion sputter coater (SPT-20, COXEM, Daejeon, Republic of Korea) at the CNU Chemistry Core Facility (Daejeon, Republic of Korea). The light transmission, reflection, and absorption of the substrates were characterized using an ultraviolet–visible–near infrared (UV–Vis–NIR) spectrophotometer (SolidSpec-3700, Shimadzu, Japan). The samples were pre-wetted by dropping water to achieve the same conditions as the absorber state during STS generation. The diffuse transmission and diffuse reflection spectra of the wet substrates were recorded via integration sphere in the wavelength range of 350 nm to 1800 nm, with a spectra resolution of 1 nm. The absorption spectra of the samples were calculated as follows:

(1) A(λ)=100−T(λ)−R(λ)

where *λ* denotes the wavelength and *T*(*λ*) and *R*(*λ*) denote the diffuse transmission and diffuse reflection of the substrates between 350 and 1800 nm, respectively. The integrated absorption values of the samples were calculated using the following equation:

(2) $$A_{solar-weight}=\int A(\lambda )\cdot W_{AM1.5G}(\lambda )\cdot d\lambda$$

where *W_AM_* 1.5*_G_* denotes the weighting factor obtained from the global air mass (*AM* 1.5*G*) condition. 

The water contact angles of the substrates were measured via a contact angle analyzer (Phoenix-10, SEO, Daejeon, Republic of Korea).

### 2.4. Solar-to-Steam Generation

STS generation was performed using a HAL-320W (Asahi Spectra, Tokyo, Japan) as the light source, calibrated for 1-sun illumination using a sun checker (CS-40, Asahi Spectra, Japan). An electronic analytical balance (ML304T, Mettler-Toledo, Columbus, OH, USA) was connected to a computer for real-time recording of weight change. The mass change of evaporated water was recorded every 10 s and averaged for every 1 min to reduce the experimental and instrumental errors. Thermal images of the absorbers were obtained using an infrared (IR) thermal camera (Testo 871). The temperature profile of each substrate was measured using the testo IRSoft Software (version 4.3). The temperature and relative humidity (RH) of the laboratory were maintained at 20 °C and 40% for all the experiments, respectively. The STS system was constructed using a water uptake design. The absorber was placed on polystyrene (PS) foam floating on the water, and the water was transported to the absorber by capillary force through a filter-paper channel. The evaporation rate of the system was calculated using the following equation:

(3) $$\dot{m}=\frac{\Delta m}{t\cdot S}$$

where Δm denotes the mass change of the system recorded by the balance over time, *t*; and *S* the evaporation surface of the absorber. 

The system efficiency was calculated using the following equation:

(4) $$\eta =\frac{\dot{m}\cdot (H_{l,v}+c\cdot (T_{S}-T_{R}))}{C_{opt}\cdot I_{0}}$$

where $\dot{m}$ denotes the evaporation rate; $H_{l,v}$ is the enthalpy of the transition process of water from the liquid to the vapor phase (2426 J·g^−1^) [53]; $T_{S}$ denotes the temperature of the absorber surface; $T_{R}$ is the room temperature; *c* is the specific heat of water (4.2 J·g·K^−1^); $C_{opt}$ denotes the optical concentration; and $I_{0}$ is the direct solar intensity of 1000 W·m^−2^.

### 2.5. SERS Experiments

R6G melamine stock solutions (10 mM) were prepared in DI water, vortexed, and sonicated until the solution became transparent. Melamine solutions with various concentrations were prepared by dilutions. The AuNP/cellulose substrates were then dipped in each solution for 1 day before analyzing the SERS spectra.

Raman spectra were obtained using a Raman microscope (LabRAM HR-800 HR Evolution HORIBA JOBIN YVON). The excitation laser wavelength was 785 nm with a 10× objective (NA:0.25) and diffraction grating of 600 gr/nm. The laser power is 228.5 µW with the spot size diameter of 3.8 µm and the confocal hole diameter is 200 µm. Before the SERS measurement, the Raman spectrometer was calibrated by a Si wafer with a characteristic Raman peak shift at 520.5 cm^−1^. The acquisition time of Raman spectra was 10 s with 3 accumulations, and the spectral resolution reached 0.77 cm^−1^. For each substrate, the Raman spectra of 5 different points were investigated all over the substrate to check the average SERS intensity of the adsorbed analyte molecules.

The limit of detection (*LOD*) in our SERS sensing data was calculated using the calibration curve of plotted SERS signal to logarithm of concentration:

(5) y=a+b⋅log(x)

where *a* and *b* are the intercept and slope of the curve, respectively.

The *LOD* SERS intensity can be calculated using the following equation:

(6) $$y_{LOD}=y_{Blank}+3SD$$

where $y_{Blank}$ is the SERS intensity of blank sample (without analyte) and *SD* is the standard deviation of the blank sample.

The *LOD* then can be calculated as:

(7) $$LOD=10^{\frac{y_{LOD}-a}{b}}$$

## 3. Results

### 3.1. Fabrication of the Substrates

**Figure 1:**
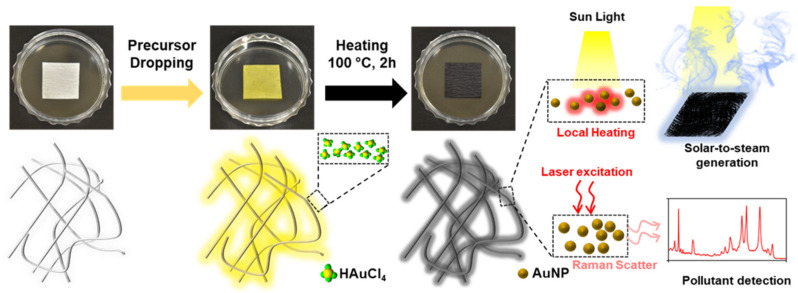
Photographs of the substrate during the fabrication process. The scheme shows the change of cellulose surface during thermal treatment. The smaller size of AuNPs will provide good absorption and the large number of AuNPs with dense nm gaps will provide strong SERS performance.

Figure 1 shows a schematic of the thermal reduction in situ fabrication of the AuNP/cellulose substrate and the possible mechanism for the dual-functional water purification and SERS detection. During thermal treatment, HAuCl_4_ was first converted to AuCl_3_, and tiny nuclei formed the Au atoms [54]. Next, the reduction and aggregation processes were continued, and Au nanoparticles were formed on the surface of the cellulose fibers [55]. The dense small nanoparticles can provide strong and broad absorption bands in the UV–Vis region, whereas the rich small nanoscale gap between the particles can provide hotspots to improve the Raman signal by increasing the electromagnetic field. The hydrophilic cellulose fibers function as the water path during the STS generation, and as a good analyte detector during SERS. 

### 3.2. Morphology and Surface Characterizations

**Figure 2:**
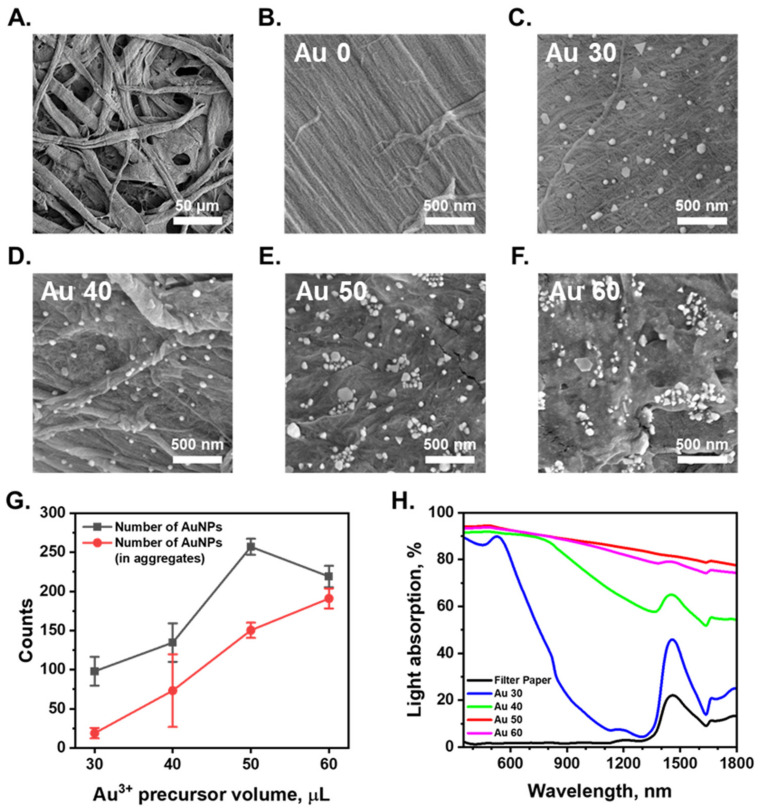
(**A**,**B**). SEM images of surface of the cellulose substrate. The bare filter paper was used as the cellulose substrate. (**C**–**F**). SEM images of AuNPs/cellulose substrates which were prepared with a range of Au^3+^ precursor volumes (30, 40, 50, 60 μL). (**G**). Number of AuNPs and number of AuNPs aggregated counted in (**C**–**F**). (**H**). Absorption spectra within sunlight wavelength range (*λ* = 350 to 1800 nm) of the wet cellulose substrate and AuNPs/cellulose substrates with different Au^3+^ precursor volumes.

Surface: The SEM images shown in Figure 2A and Figure S1 indicate that the filter paper substrate and AuNPs/cellulose substrate have porous structures with micrometer-size pores and several layers of fibers. This porous structure can provide a good pathway for evaporated vapor escape, and improve the energy transfer efficiency by interfacial local heating, thus providing efficient conversion of solar energy into heat energy, which can be utilized for water purification. The hydrophilicity of filter paper and AuNPs/cellulose substrate were characterized via contact angle measurement, as shown in Figure S3. The water was quickly absorbed through the surface of both cellulose filter paper within 0.1 s and the AuNPs/cellulose substrate within 1 s. This observation suggests that the surface of the AuNPs/cellulose substrate is more hydrophobic than the bare paper, but still had high hydrophilicity, making it a good water evaporation surface.

The number of plasmonic nanoparticles and their distribution on the substrate is an essential factor to be considered in optimizing the SERS performance of the substrate. An increase in the number of AuNPs grown on the fiber surface of the AuNPs/cellulose substrate can increase the number of NP aggregates with small nm gaps and the number of NPs in the aggregates, which in turn increases the SERS performance of the substrate. Applying a higher amount of the Au precursor onto the fiber surface (higher volume of the precursor solution) can increase the number of AuNPs and the aggregates on the fiber surface, resulting in a stronger SERS performance of the substrate. For a better understanding of the formation of AuNPs on the cellulose substrate, we conducted SEM analysis of all the substrates made with different Au^3+^ precursor volumes of 30 µL (Au 30), 40 µL (Au 40), 50 µL (Au 50), and 60 µL (Au 60). The substrates made with low precursor volumes of 30 µL and 40 µL showed a small number of AuNPs, with very few aggregated structures, as can be seen in Figure 2C,G. The gaps between these individual particles are more than a hundred nanometers and cannot work as SERS hot spots. With the precursor volume of 50 or 60 µL, the surface morphologies of the substrates are quite similar. A large number of AuNPs were formed on the same surface, which is clearly demonstrated in Figure 2E–G. A histogram showing the particle sizes distribution of sample Au 50 presented in Figure S2 demonstrates that the average size of AuNPs is 56 ± 20 nm. The larger number of aggregated structures that appeared increased the number of nanoscale gaps between the particles, ultimately creating numerous hot-spot regions that can provide a strong SERS signal of the analytes adsorbed on this site. With a high number of AuNPs and the aggregated structures all over the substrate surface, the substrates made with precursor volumes of 50 µL or 60 µL will be better for SERS performance.

Light absorption: UV–Vis spectrophotometry was conducted to qualitatively assess the AuNP/cellulose absorbers. Some portion of the light could be absorbed or reflected by the wet cellulose filter paper. For clarity, we provide the optical properties of the wet cellulose filter paper. The wet cellulose filter paper does not absorb the light at the UV-Vis spectra range (*λ*: 350–700 nm). The absorption band of wet cellulose paper is near *λ* = 1500 nm, which is related to the absorption band of water. In comparison, the dense layers of gold nanoparticles absorption bands clearly overlap with the cellulose absorption band. The light interaction of AuNPs is critical in the characterization of the optical properties of the AuNPs/cellulose substrate. The light absorption spectra of the AuNP/cellulose substrates are shown in Figure 2H. The substrate Au 30 showed a high absorption property in the UV-VIS range, with a characteristic peak of AuNPs at 520 nm. As the volume of the Au^3+^ precursor increase, the number of individual AuNPs and aggregated AuNPs also increases, as can be seen in Figure 2G. The larger number of aggregates leads to the appearance of larger number of nm-gap SERS hotspots, so the absorption ranges of the substrate were broadened and the absorption intensity also increases. The latter statement is apparent from the comparison of the difference in the absorption spectra in the NIR region between the sample Au 30 and Au 40. The substrates Au 50 and Au 60 exhibited 90% broadband absorption between 350 and 1800 nm, with a maximum absorption of 95% in the visible region and minimum absorption of 78% (Au 50) and 75% (Au 60) in the NIR region. In addition, the SERS intensity of the substrate was related to the improvement of the electromagnetic field intensity in the laser and Stokes shift wavelengths. In this work, we used the excitation of 785 nm for all the SERS measurements, and the Stokes shift wavelengths were also in the NIR region. The AuNPs-cellulose substrates with the Au^3+^ precursor volumes of 50 µL and 60 µL, showing high absorption value in the NIR region, could provide good SERS intensity, making them good SERS substrates.

### 3.3. Solar-to-Steam Generation Performance

**Figure 3:**
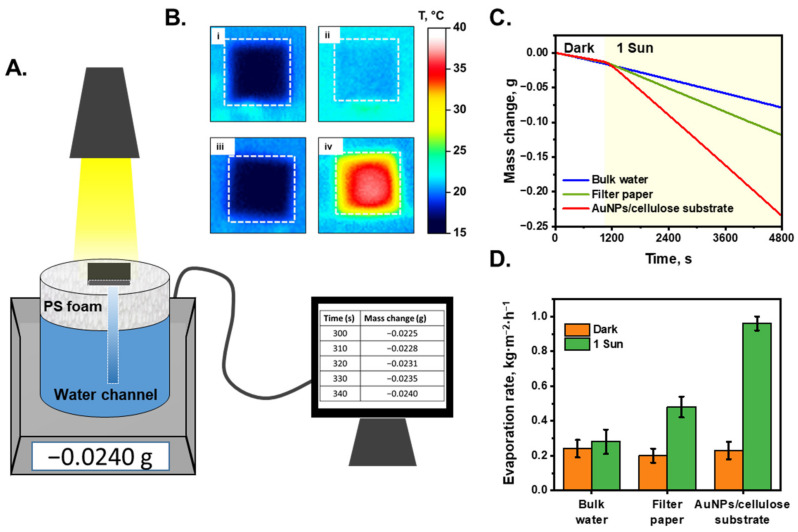
Solar-to-steam generation performance. (**A**). Scheme of the evaporation system. The mass change was recorded by electronic balance every 10 s. (**B**). (**i**,**ii**): The temperature profile of the wet filter paper under dark condition and 1-sun illumination, respectively; (**iii**,**iv**): the temperature profile of the wet black absorber under dark condition and 1-sun illumination. The white dashed boxes denote the size of the substrate. (**C**). Mass change of the systems with different light absorbers. The solar light was turned on after 20 min working in dark conditions. (**D**). Corresponding evaporation rate of each system under dark condition and under 1-sun illumination.

Figure 3A shows a schematic of the STS system. In this study, the purification performance of an interfacial evaporation system was characterized under dark and 1-sun illuminated conditions. The AuNP/cellulose substrate was placed on top of the PS foam, and water was absorbed using a cellulose channel, which was connected to the substrate through a hole in the PS foam layer. The mass change of the system was recorded as the mass of the evaporated water. Figure 3B(i,ii) show the temperature profile of the white cellulose substrate and AuNP/cellulose substrate as the absorber under dark and 1-sun illuminated conditions. The temperature of the cellulose substrate under these conditions was not significantly different (~3 °C), which can be explained by the fact that the white substrate cannot absorb the light energy efficiently, and most of the energy was lost to the environment by reflection; thus, the energy provided to the water was not sufficiently high and the water temperature did not increase significantly. However, when the AuNP/cellulose substrate functioned as an absorber with significant light absorption, the light energy was received and converted to heat energy by Landau damping [56], and then transferred to the water that covered the AuNPs; therefore, the temperature of the AuNP/cellulose under 1-sun illumination was significantly higher (32.5 °C) than that under dark conditions (18 °C), as can be seen in Figure 3B(iii,iv). The STS generation performance of the AuNPs/cellulose substrates with different loading volumes of Au^3+^ precursor was shown in Figure S4. As expected, the substrate Au 50, which shows the highest light absorption value, also provides the highest water evaporation rate, as compared to Au 30, Au 40 and Au 60. Thus, the Au 50 substrate will be utilized for further characterization of STS experiment and also for the SERS performance. Figure 3C shows the mass change of the evaporation system with each absorber, and the evaporation rate of each system was determined using Equation (3) (Figure 3D). Under dark conditions, the evaporation rate of the bulk water was 0.24 kg m^−2^ h^−1^, water in the cellulose substrate was 0.2 kg m^−2^ h^−1^, and that of water in the AuNP/cellulose substrate was 0.23 kg m^−2^ h^−1^, all of which are similar. When the solar simulator was turned on and the illumination power was 1 sun, because the water does not absorb the light energy to convert to heat energy, the evaporation rate of the bulk water was 0.28 kg m^−2^ h^−1^, which is similar to the evaporation rate under dark conditions. The evaporation rate of the cellulose substrate under 1 sun was 0.5 kg m^−2^ h^−1^, which can be explained by the fact that the water cover layers on the cellulose surface are thinner than bulk water; thus, the thermal loss is lower, and more heat is used for water evaporation. When the absorber was changed to the AuNP/cellulose substrate, the evaporation rate under 1-sun increased to 0.96 kg m^−2^ h^−1^, which is nearly double that of the evaporation rate of the cellulose substrate. Based on Equation (4), the evaporation efficiency of the system using the AuNP/cellulose substrate was calculated to be 67%. In STS generation, stability is also a very important factor for evaluating the system performance. Therefore, a 30 h continuous STS experiment under 1-sun illumination was applied to perform the stability property of the AuNPs/cellulose substrate. As can be seen in Figure S9, there was no significant decay observed in the water evaporation rate of the system. This indicated that the AuNPs/cellulose substrates are stable for STS generation applications.

### 3.4. SERS Performance

To demonstrate the chemical detection performance of the AuNP/cellulose substrate, the common Raman molecule, R6G, and a model analyte, melamine, an illegal food contaminant, were used. To quantify the contribution of AuNPs in our AuNPs/cellulose substrate, the Raman spectra of R6G with a concentration of 100 µM were obtained using the Au 50 substrate and bare cellulose substrate, in the same measurement parameter. Figure S7 demonstrates that the strong SERS signals were provided by the AuNPs on the Au 50 substrate, while the bare cellulose paper did not show any fingerprint spectra of R6G. A similar examination was performed using melamine at the concentration of 1 mM, as shown in Figure S8. These results prove that a strong SERS signal is mainly attributed to plasmonic nanoparticles and not the cellulose backbone.

**Figure 4:**
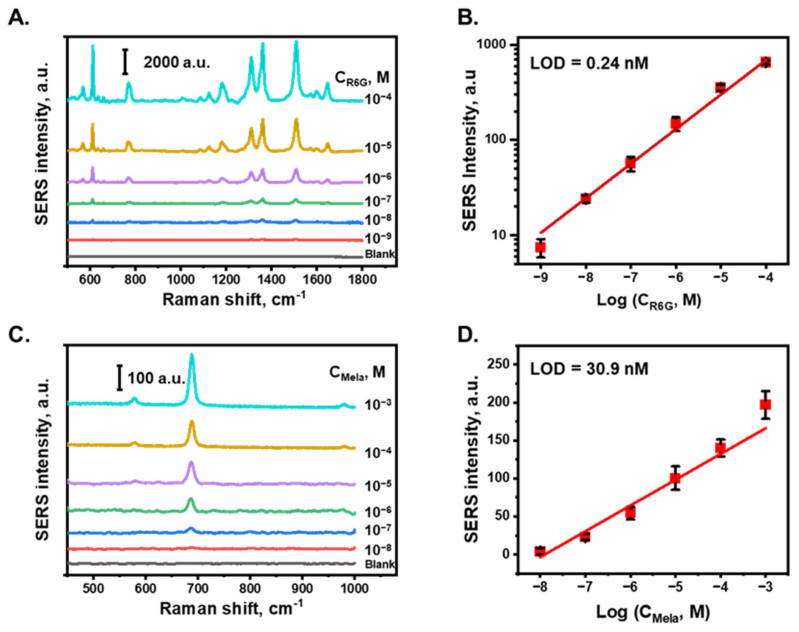
SERS detection performance. (**A**). SERS spectra of R6G at various concentrations from 100 µM to 1 nM in DI water, obtained with AuNPs/cellulose substrate. (**B**). Calibration plot of SERS intensity of R6G with peak at 611 cm^−1^. (**C**). SERS spectra of melamine at various concentrations from 1 mM to 10 nM in DI water. (**D**). Calibration plot of SERS intensity of melamine.

Figure 4A shows the SERS spectra of R6G in DI water at various concentrations. The characteristic peaks of R6G at 612, 768, 1180, 1311, 1360, 1509, and 1646 cm^−1^ were attributed to the C–C–C ring in-plane vibration mode, C–H out-of-plane bending mode, C–H in-plane bending mode, N–H in-plane bending mode, and C–C stretching mode of the R6G molecule, respectively [57]. Figure 4B shows the relationship between the SERS intensity of the peak at 618 cm^−1^ and the logarithm of the R6G concentration, which exhibited a linear relationship with a correlation coefficient of 0.99 between 10^−9^ to 10^−4^ M. For each concentration sample, the SERS intensities from five different measured points were averaged to obtain the SERS intensity of this concentration. The raw data measurement of R6G and melamine are shown in Figures S5 and S6, respectively. The limit of detection (LOD) of R6G was calculated based on three times the standard deviation of the blanks divided by the slope of the calibration curve and was determined to be 0.24 nM in DI water. In this study, the SERS detection performance of an AuNP/cellulose substrate with melamine, which is an illegal chemical that is typically added to food to increase the protein content [58,59], was also characterized. Figure 4C shows the SERS spectra of melamine at different concentrations in DI water. The characteristic Raman band of melamine at 681 cm^−1^ was attributed to the in-plane deformation of the triazine ring breathing mode, primarily from carbon–amine bonds [60]. The SERS intensities of the peak at 681 cm^−1^ for different melamine concentrations are plotted in Figure 4D, with the *x*-axis denoting the logarithm of the concentration. A linear relationship was obtained with a correlation coefficient of 0.97 between 10^−8^ to 10^−3^ M. Based on the relative standard deviation value of blank noise, the LOD value of melamine in DI water was determined to be 30.9 nM, which is four orders of magnitude lower than the allowed level of melamine by the World Health Organization and US Food and Drug Administration [52]. These results indicate that the substrate can perform well as a SERS platform to detect trace chemicals in water.

### 3.5. Dual-Functional Performance

**Figure 5:**
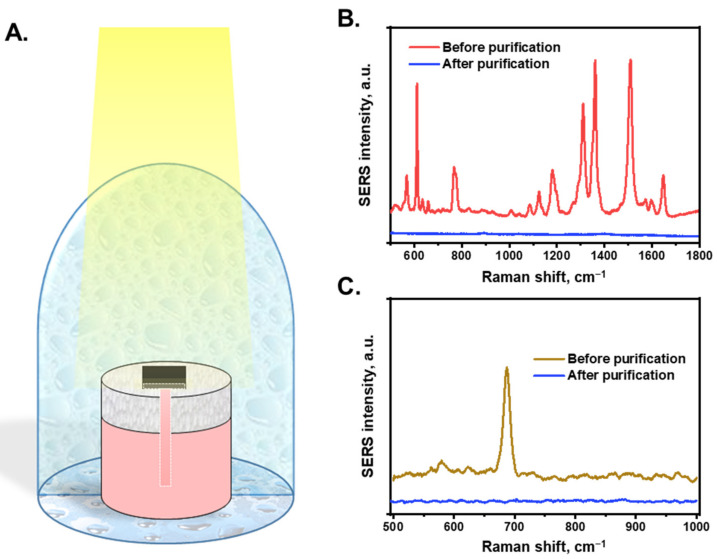
(**A**). The schematic shows the contaminated water purification and pollution detection using AuNPs/cellulose substrate. The water after purification will be collected and detected again to check the quality. (**B**). The SERS intensity of R6G-contaminated water (10^−5^ M) before and after purification process. (**C**). The SERS intensity of melamine-contaminated water (10^−5^ M) before and after purification process.

By combining STS generation and SERS sensing functionalities, the AuNP/cellulose substrate was employed for the on-site purification and detection of water. Figure 5A shows a schematic of the water purification and detection system using an AuNP/cellulose substrate. The system was covered with a clean glass dome to create a closed system. First, the substrate analyzed polluted water in the tank using SERS. Subsequently, the water was purified using STS generation. The water droplets condensed on the glass were collected as purified water and then reanalyzed by SERS. As the source, the water containing 10^−5^ M R6G was kept in the tank before purification, and the SERS spectra are shown in Figure 5B. Next, the solar water purification and collection were performed, and the SERS spectra of the water were compared with the polluted water. As shown in Figure 4A, the R6G dye at a concentration <10^−8^ M in water was clearly detected by SERS. Figure 5B demonstrates that the SERS spectrum of purified water did not exhibit any peaks, whereas the R6G peaks were clearly appeared before purification. Melamine (10^−5^ M) was characterized using the same process (Figure 5C). Before purification, the SERS spectra of melamine clearly exhibited a peak at 681 cm^−1^. However, after purification, this peak could not be clearly observed, even though the substrate could detect melamine at 10^−7^ M (Figure 4C). This indicates that the R6G dye and melamine contained in the water after purification were at a lower concentration than the requirement of clean water, which is safe for drinking.

## 4. Conclusions

A dual-functional substrate for STS generation and SERS detection of water impurities was successfully prepared by thermally reducing AuNPs on cellulose substrate. The substrate demonstrated the possibility of functioning as a dual-functional platform as a STS generator to purify polluted water as well as a SERS sensor to detect trace chemicals in water. The substrate exhibited a 90% broadband light absorption in visible light, with a porous cellulose fiber matrix that provides a good pathway for water, thus rendering it a good absorber to function as an evaporation surface in solar water purification. The substrate can also function as a SERS substrate to enable ultrasensitive detection of R6G and melamine in DI water down to the nanomolar level. An onsite experiment demonstrated the dual-function performance of the substrate. The polluted water was investigated to identify the pollution chemical, after which the water was purified and reinvestigated for accuracy, thus proving the capability of the dual-functional properties of the AuNP/cellulose substrate.

## Data Availability

Data are available in the main text.

## Supplementary Materials

The following supporting information can be downloaded at: https://www.mdpi.com/article/10.3390/nano13061003/s1, Figure S1. SEM EDS analysis of cross-section cellulose filter paper and AuNPs/cellulose substrates; Figure S2. The size distribution of AuNPs on the AuNPs/cellulose substrate; Figure S3. The water contact angle measurement; Figure S4. The solar-to-steam generation performance of AuNPs/cellulose substrate; Figure S5. Raw data measurement of R6G dye; Figure S6. Raw data measurement of melamine in DI water; Figure S7. SERS performance of the bare cellulose paper and the AuNPs/cellulose substrate with the R6G dye; Figure S8. SERS performance of the bare cellulose paper and the AuNPs/cellulose substrate with the melamine; Figure S9. The 30 h continuous working of the AuNPs/cellulose substrate in the purification system under 1-sun illumination.
