# Supplementary material for: Dual-Functional Solar-to-Steam Generation and SERS Detection Substrate Based on Plasmonic Nanostructure
Source: Nanomaterials (Basel). 2023 Mar 10;13(6):1003. doi: 10.3390/nano13061003 (PMC10054297; doi:10.3390/nano13061003)
Supplement: Supplementary file 1 [file nanomaterials-13-01003-s001.zip › nanomaterials-2225687-supplementary.pdf]

Supplementary Materials

Dual-Functional Solar-to-Steam Generation and SERS Detection Substrate Based on Plasmonic Nanostructure

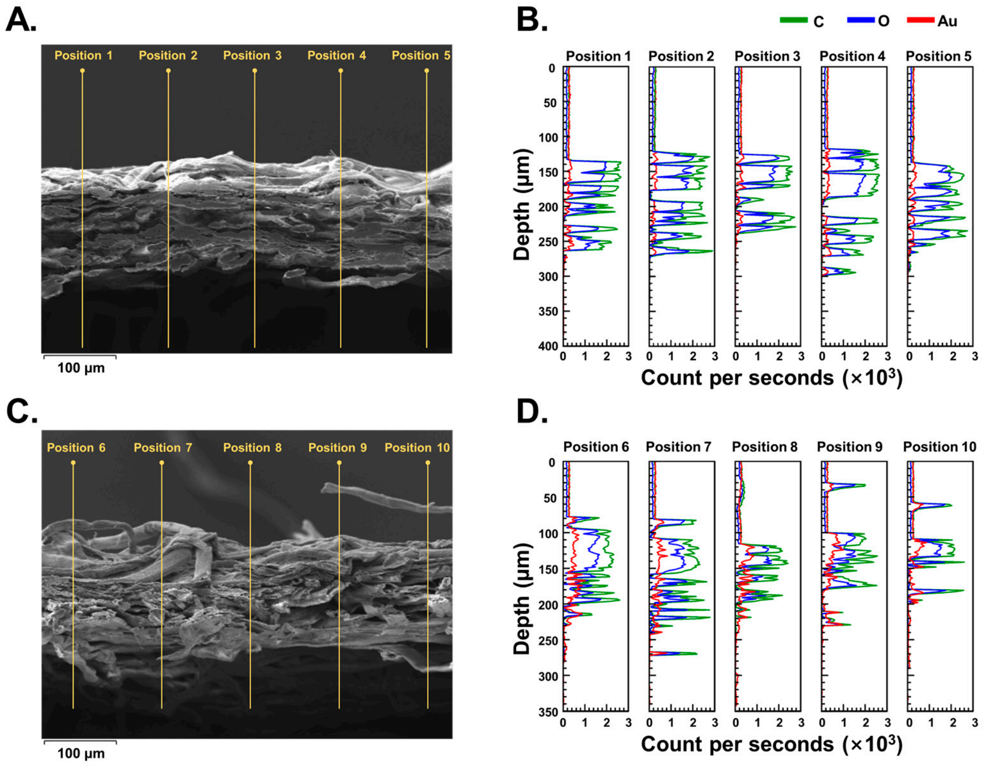

Figure S1. SEM EDS analysis of cross-section cellulose filter paper and AuNPs/cellulose substrate. A. Cross-section of cellulose filter paper. The EDS line scan analysis over the cellulose cross-section showing in B. indicated the appearance of carbon and oxygen elements, which come from cellulose fibers, there is no appearance of gold observed. C. The cross-section of AuNPs/cellulose substrate. D. The EDS line scan analysis over the AuNPs/cellulose substrate shows the appearance of Au together with the carbon and oxygen. Pt was deposited on filter paper and AuNPs/cellulose substrate for charge reduction and protection of SEM samples, respectively.

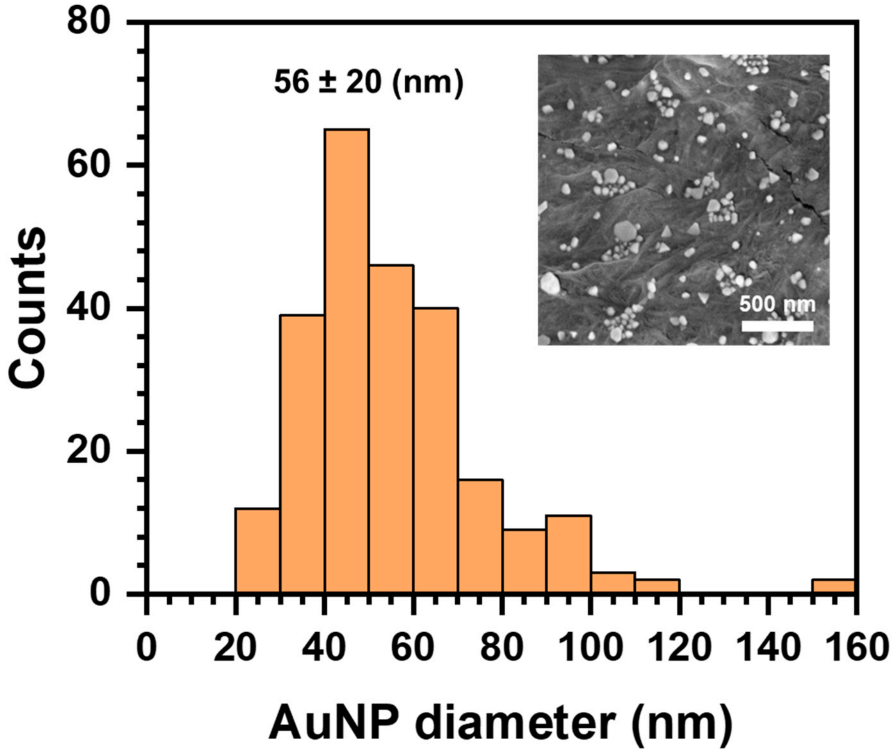

Figure S2. The size distribution of AuNPs on the AuNPs/cellulose substrate prepared with the Au^{3+} precursor volume of 50 μL.

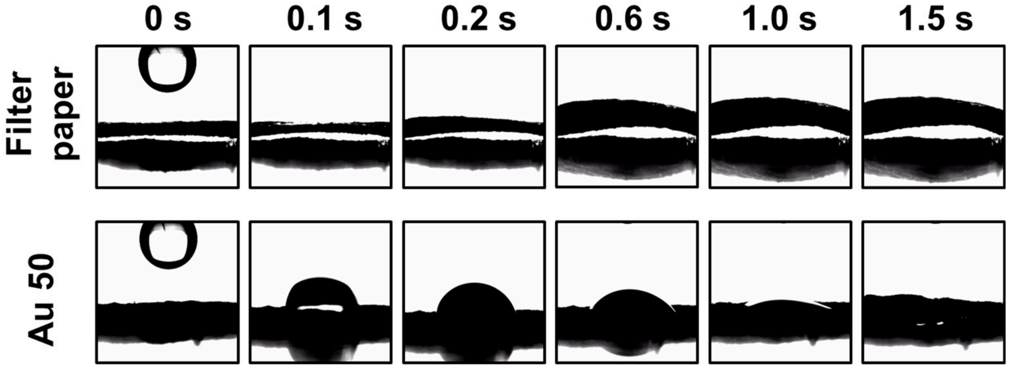

Figure S3. The water contact angle measurement of cellulose substrate and the AuNPs/cellulose substrate made with the Au^{3+} precursor volume of 50 μL.

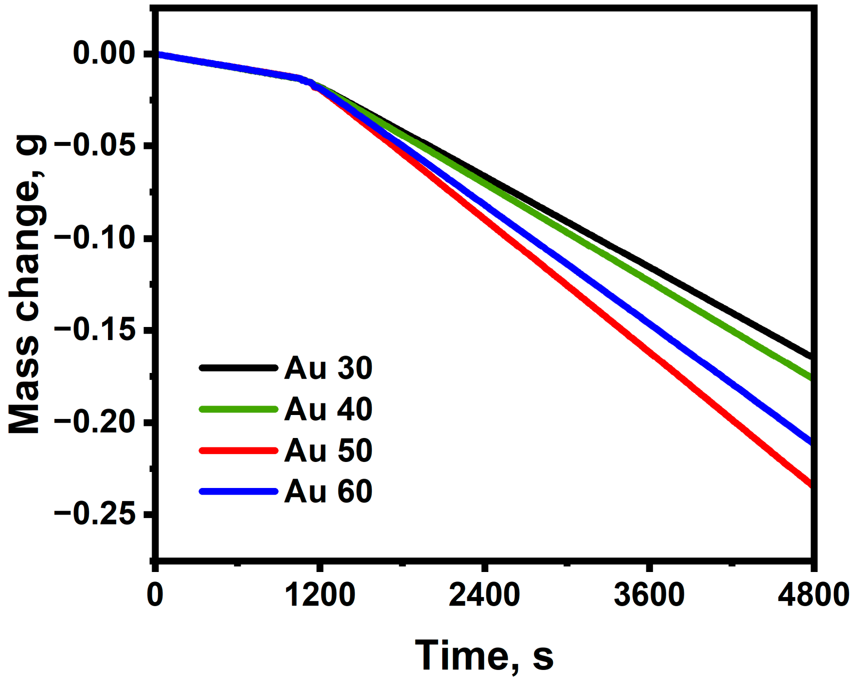

Figure S4. The solar-to-steam generation performance of AuNPs/cellulose substrate fabricated with different Au^{3+} precursor volumes.

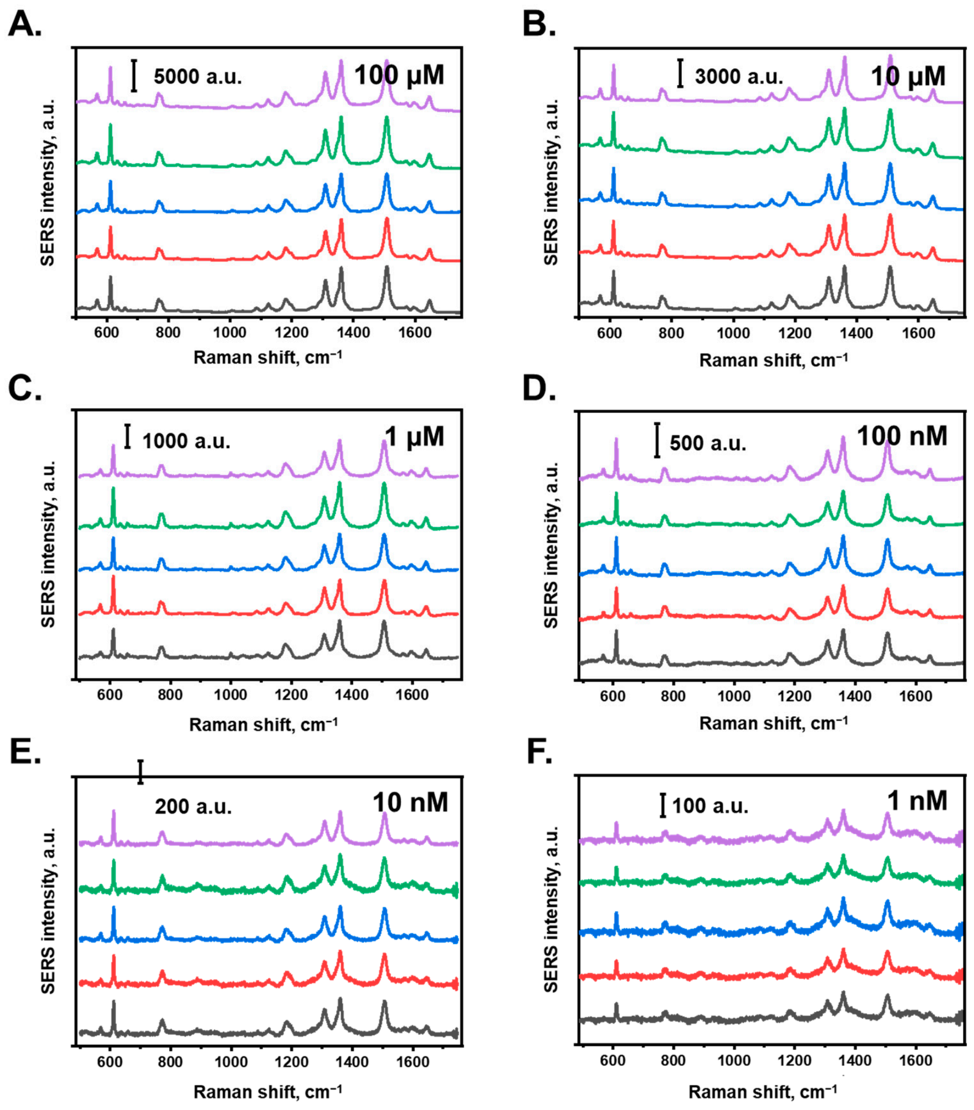

Figure S5. Raw data measurement of R6G dye with a range of concentration from 100 μM to 1 nM (A–F).

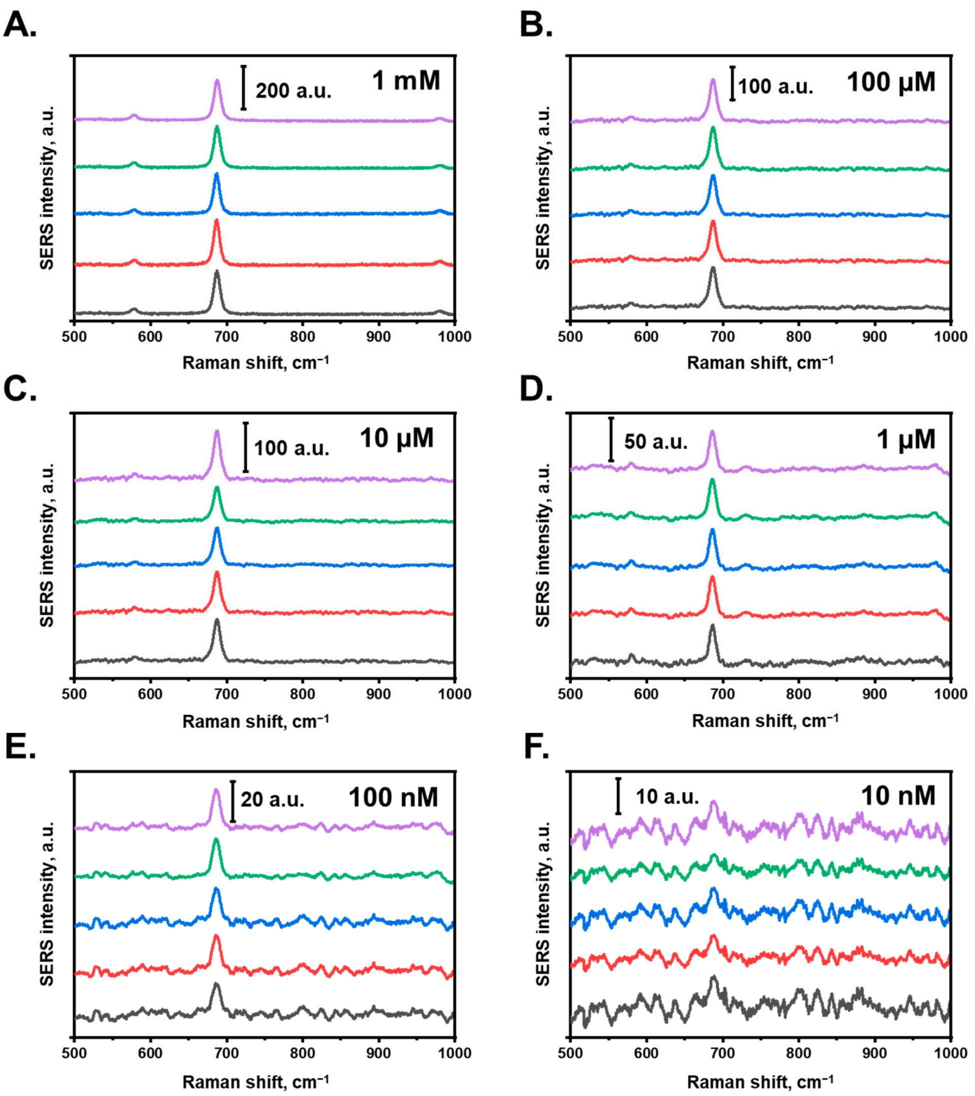

Figure S6. Raw data measurement of melamine in DI water with a range of concentration from 1 mM to 10 nM (A–F).

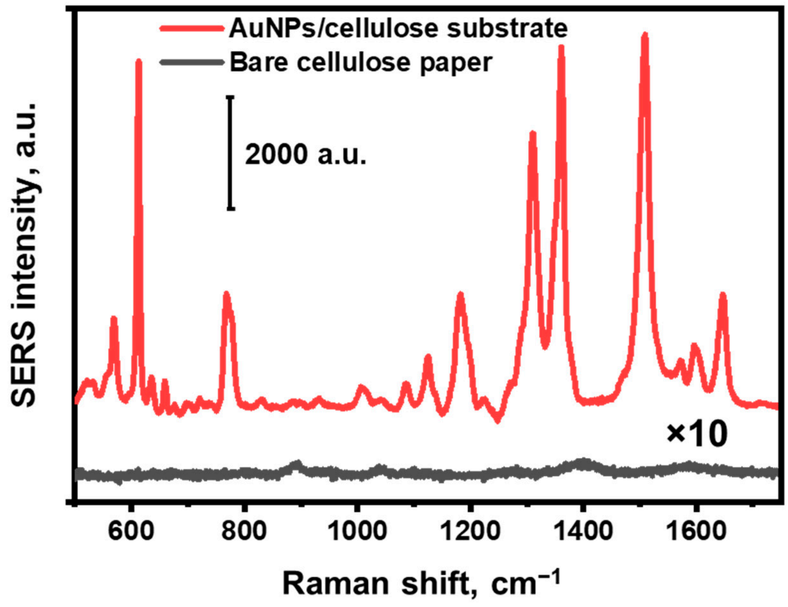

Figure S7. SERS performance of the bare cellulose paper and the AuNPs/cellulose substrate with the R6G dye at the concentration of 100 μM. The Raman spectrum collected by bare cellulose paper was enlarged multiple 10 times.

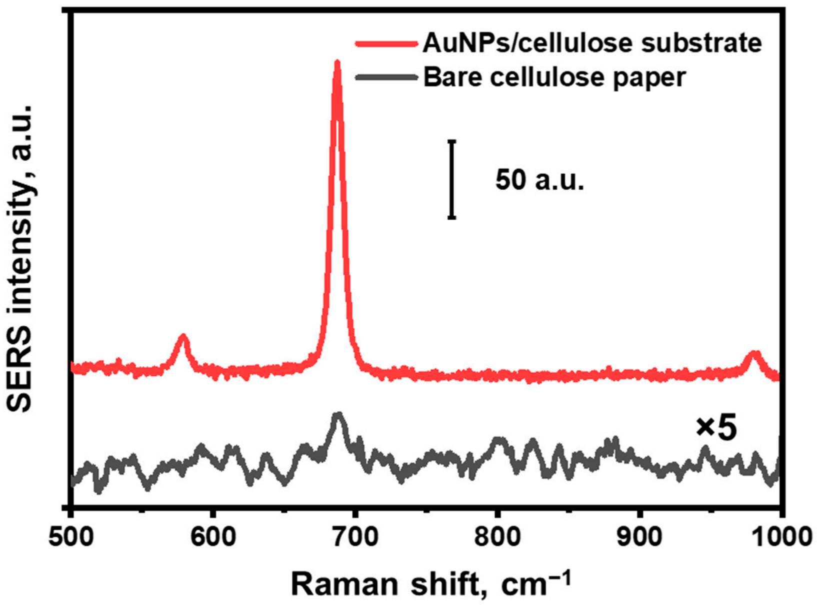

Figure S8. SERS performance of the bare cellulose paper and the AuNPs/cellulose substrate with the melamine at the concentration of 1 mM. The Raman spectrum collected by bare cellulose paper was enlarged multiple 5 times.

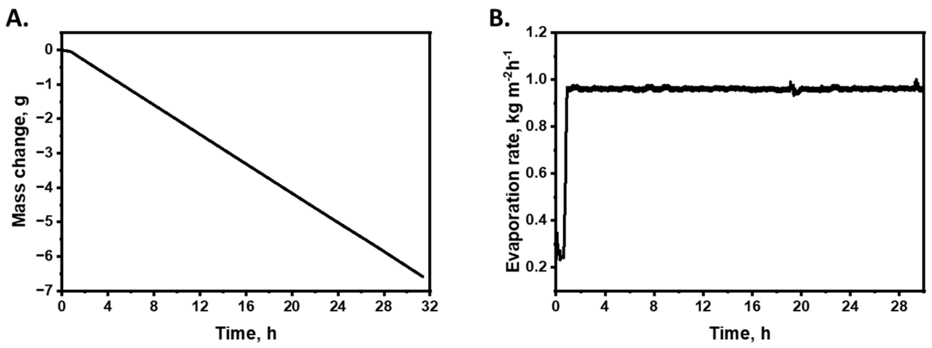

Figure S9. The 30-hour continuous working of the AuNPs/cellulose substrate in the purification system under 1 Sun illumination. A. The evaporated water mass recorded during the evaporation process and B. The evaporation rate of the system.
